# Barriers on cross-border sharing of health data for secondary use and options to overcome these

**DOI:** 10.1093/eurpub/ckac129.367

**Published:** 2022-10-25

**Authors:** R Richards

**Affiliations:** European Digital and Research Policy, NHS Confederation, Brussels, Belgium

## Abstract

Cross-border health data sharing is essential to facilitate vital health research into new treatments and to improve patient care and safety across Europe. The flagship European Health Data Space (EHDS) aims to promote better exchange and access to health data through the creation of a European-wide infrastructure. However, significant barriers to cross-border data sharing must first be resolved in order to fully ensure the successful functioning of the Data Space. To this end, the European Commission established a Joint Action (TEHDAS), as the policy development tool for the development of the EHDS. This presentation will be based around the findings of the recent TEHDAS report ‘Barriers to cross-border sharing of health data for secondary use and options to overcome them.’ The report draws on the results of a dedicated literature review, 133 case studies from across 23 countries and expert interviews to identify the primary barriers to cross-border data sharing and options to overcome these from the perspective of European data users (research and policymakers). The session will start with a concise presentation of the report methodology. The research team will then deliver an interactive presentation, in which they succinctly outline each barrier and policy option and ask participants to vote for their preferred policy option to overcome each barrier identified. EUPHA participants votes will inform the final TEHDAS recommendations on mitigations and solutions for barriers to cross-border data sharing which will be submitted to the European Commission for incorporation in the European Health Data Space legislative proposal.

